# Evaluation of Neck Disability Using Computed-Tomography in Head and Neck Cancer Survivors

**DOI:** 10.3389/fpain.2022.910247

**Published:** 2022-05-17

**Authors:** Alexandria Harris, Barton Branstetter, Jinhong Li, Sara R. Piva, Jonas T. Johnson, Marci Lee Nilsen

**Affiliations:** ^1^Department of Otolaryngology, University of Pittsburgh School of Medicine, Pittsburgh, PA, United States; ^2^Department of Radiology, University of Pittsburgh School of Medicine, Pittsburgh, PA, United States; ^3^Department of Biostatistics, School of Public Health, University of Pittsburgh, Pittsburgh, PA, United States; ^4^Department of Physical Therapy, University of Pittsburgh School of Health and Rehabilitation Sciences, Pittsburgh, PA, United States; ^5^Department of Acute and Tertiary Care, School of Nursing, University of Pittsburgh, Pittsburgh, PA, United States

**Keywords:** head and neck cancer, pain, computed tomography, survivorship, neck disability

## Abstract

**Objective:**

We have previously reported that 55% of head and neck cancer survivors have neck disability. However, it is unclear what factors contribute to their neck disability. Our study aim is to determine if survivors with neck disability have evidence of cervical spine degenerative disease assessed by computed tomography (CT).

**Materials/Methods:**

Cross-sectional analysis of patient-reported neck disability, prospectively collected on survivors of squamous cell carcinomas without recurrence or metastasis over one-year post-treatment. Neck disability and its impact on daily life was measured using the Neck Disability Index (NDI) and compared with cervical CT scans within 6 months. Scans were evaluated for degeneration of the disc and facet of the cervical vertebrae rated on a 5-point scale where 5 indicates more severe disease. Multivariable linear regression was used to analyze the association between NDI and radiographic findings.

**Results:**

116 survivors of oropharyngeal carcinomas were identified, predominantly male (81.9%) with an average age of 62.8 ± 8.2 (range 43.8–81.4). Most survivors had advanced stage III-IVa cancer (94.0%) with treatment modalities including surgery (n=26, 52.0%), chemotherapy (*n* = 45, 90.0%), and radiation therapy (*n* = 49, 98.0%). Absence of neck disability was observed in 44.0% of survivors, 39.7% had mild disability, and 16.4% moderate disability. The time from treatment to clinic visit was an average of 3.1 ± 2.7 years (range 1.1–13.4). Multivariable analysis of NDI controlling for age, time since treatment, and treatment modality identified an inverse association between NDI and spinal degenerative disease examining cervical discs (−1.46 95% confidence interval (CI) [−2.86, −0.06], *p* = 0.041) and age (−0.24 95% CI[−0.40, −0.08], *p* = 0.004).

**Conclusions:**

Our study shows that neck impairment and pain in head and neck cancer survivors is not sufficiently explained by cervical degeneration related to age or trauma, supporting the theory that post-treatment neck disability occurs as a side effect of treatment. These results support the further assessment of structure and function of cervical musculature and degeneration following HNC treatment.

## Introduction

Head and neck cancer (HNC) is one of the most common cancers worldwide (1). Advancement in HNC management including the incorporation of multimodal therapy such as surgical resection and chemoradiation has led to improved survival among those affected by the disease. Increases in human papillomavirus (HPV)-associated squamous cell oropharyngeal cancers have been identified with the demographics of patients shifting to a younger cohort (2–6). While HNC numbers have remained stable as tobacco use becomes less common, this transition has led to a younger HNC population with extended survivorship courses. These survivors often experience complex, often unpredictable survivorship courses due to the late- and long-term effects of treatment associated with the intensification of therapy and impact on psychosocial well-being (7). While intensification of treatment has contributed to an increased quantity of life, it does not necessarily result in an increased quality of life.

One of the most common side effects of treatment is neck disability, impaired mobility and pain years after treatment completion, affecting up to 70% of patients (8–10). All therapy modalities for HNC can contribute to this disability, with surgical intervention leading to disruption of cervical muscles and scarring and radiotherapy contributing to soft tissue and muscle fibrosis (11–13). These deficits often result in substantial detriments to the quality of life of survivors with symptoms increasing over time (14–16). Given the prevalence of neck disability in the HNC population, there is a growing need to understand the relationship between patient reported symptoms and pathophysiology of neck disability.

Currently, subjective reports and patient-reported outcome (PRO) measures are used to assess the functional deficits faced by survivors. This includes previously published research on the usefulness of the Neck Disability Index, a tool which examines neck disability, pain, and reduced range of motion in patients having undergone treatment (17). Additional objective measurements of neck impairment in cervical range of motion and velocity have been noted in survivors who report neck disability, but is limited in application to the literature and by the use of time-consuming sensor and data processing (18). To further assess the physical implications of reported neck disability and to target survivors with ongoing healthcare needs, there is a need for additional objective measures.

Our study evaluates the relationship between reported neck disability and findings on radiographic imaging to understand the interaction between reported neck impairment and anatomical changes seen in imaging. The primary aim of our study is to examine the relationship of the NDI with neck CT scans obtained after treatment in survivors of HNC.

## Methods

We conducted a cross-sectional analysis of patient-reported neck disability symptoms, prospectively collected on survivors of squamous cell carcinomas without recurrence or metastasis evaluated in a multidisciplinary head and neck survivorship clinic from January 2017 to September 2020. **Survivors who completed the NDI within 6 months of a cervical CT were included**. Those who were <1-year post-treatment were excluded to minimize the impact of acute treatment toxicity.

### Demographics and Clinical Characteristics

Demographics and clinical characteristics were abstracted from the medical record. Variables obtained included: age, gender, race, marital status, tumor site, American Joint Committee on Cancer (7^th^ and 8^th^ Edition) staging, treatment modality (i.e., surgery alone, non-surgical radiation and/or chemotherapy, or surgery with adjuvant), and time since treatment completion.

### Neck Disability Index

Neck disability was assessed using the Neck Disability Index (NDI), a 10-question measure of disability resulting from neck pain with higher scores indicating more severe disability as recommended for HNC patients by Vernon and Mior (19) and Spinelli et al. (20). Questions evaluate the impact pain has on functional activities such as personal care, sleep, and movement. Each item is scored from 0 to 5, with total scores ranging from 0 to 50 (19). For this analysis, NDI was categorized into three groups: no disability (≤4), mild disability (5–14) or moderate disability (≥15). Previous studies in mechanical neck disorders, musculoskeletal dysfunction, cervical radiculopathy, and non-specific neck pain have identified an approximate 10-point score change as the minimal detectable change needed to ensure observations are relevant (21–24). The NDI has been used to measure neck dysfunction with reliability (r = 0.890) and internal consistency (∝ 0.890–0.920) in HNC patients along with self-reported improvement in activity following treatment (8, 19, 25–28).

### CT Evaluation

**Figure 1:**
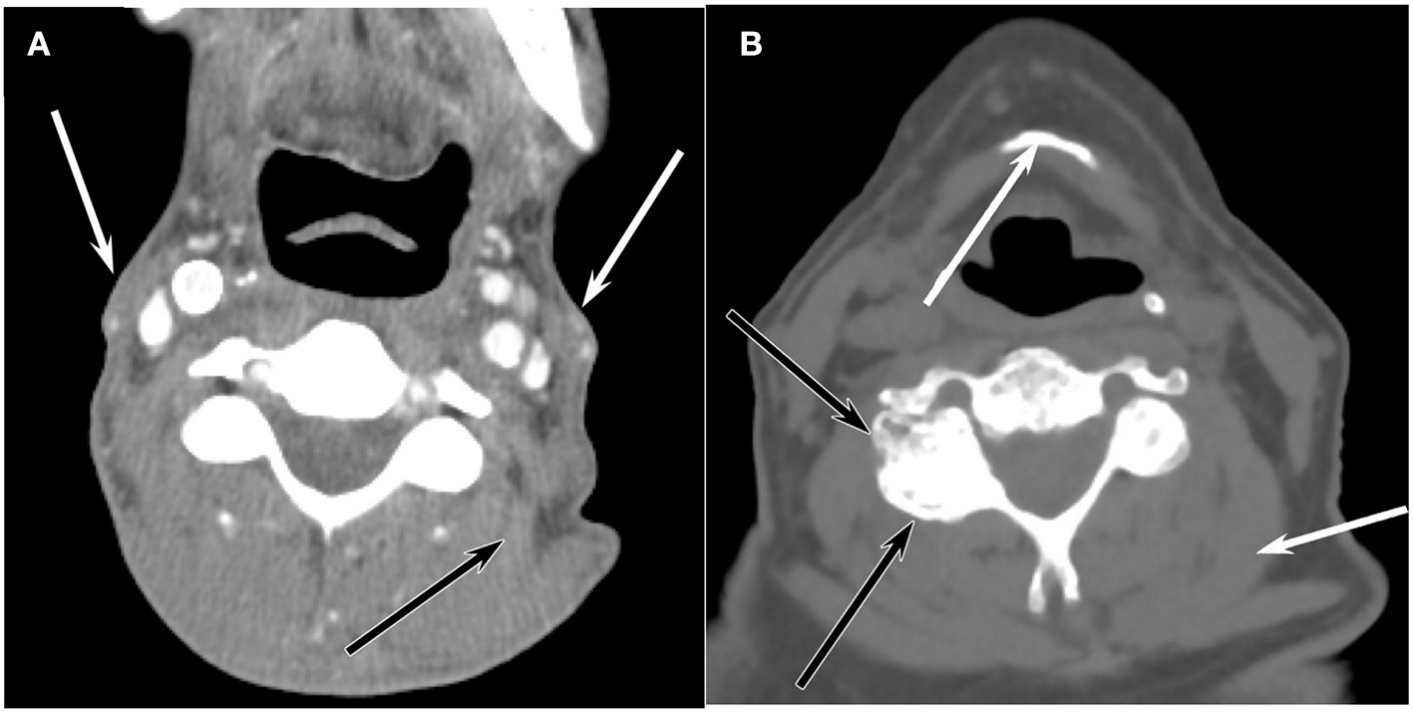
Axial CT representations of **(A)** Enhanced axial CT of a 56-year-old man at the level of C3 shows marked atrophy of the sternocleidomastoid muscles (white arrows) and paraspinal muscles such as the levator scapulae muscle (black arrow), but there is no appreciable degenerative disease in the facet joints or discs. **(B)** Unenhanced axial CT image at the level of C3 reveals extensive facet arthrophy (black arrows) and disc disease, but the muscle volume (white arrow) is normal for a 71-year-old man.

Neck CT imaging performed in routine cancer surveillance was identified for survivors. Scans within 6 months of a survivor NDI survey were used for the study. CT scans were performed on GE Lightspeed systems with 64 data channels. Acquisition parameters included: 100 kVp, automated mA (generally 100–200), FOV 18–25 cm, matrix 512 × 512, pitch 0.53. Axial 1.25 mm-thick reconstructions were created with bone and soft tissue kernels, which were then reformatted into sagittal and coronal planes. Images were evaluated by a head and neck radiologist with 20 years of experience in practice blinded to neck disability scores and patient characteristics. The degree of degeneration was categorized based on subjective analysis of the entire neck rather than relying on a single cervical level since degenerative disease and muscle loss are often non-uniform through the cervical spine. Disc and facet degeneration were rated on a 5-point scale (1 = none, 2 = mild, 3 = moderate, 4 = severe, 5 = complete disease) with examples seen in Figure 1.

### Statistical Analysis

Statistical analysis was performed using RStudio (1.1.456; RStudio, Inc, Boston, Massachusetts). Mean and standard deviation were calculated for continuous variables; frequency and percentage were calculated for categorical variables. Fisher's Exact test was used to examine the differences between demographic and clinical characteristics from the NDI, disc, facet, scores. Controlling for age, time since treatment, and treatment modality, multivariable regression analysis was performed to investigate the association between neck disability, disc, and facet degeneration.

## Results

**Figure 2:**
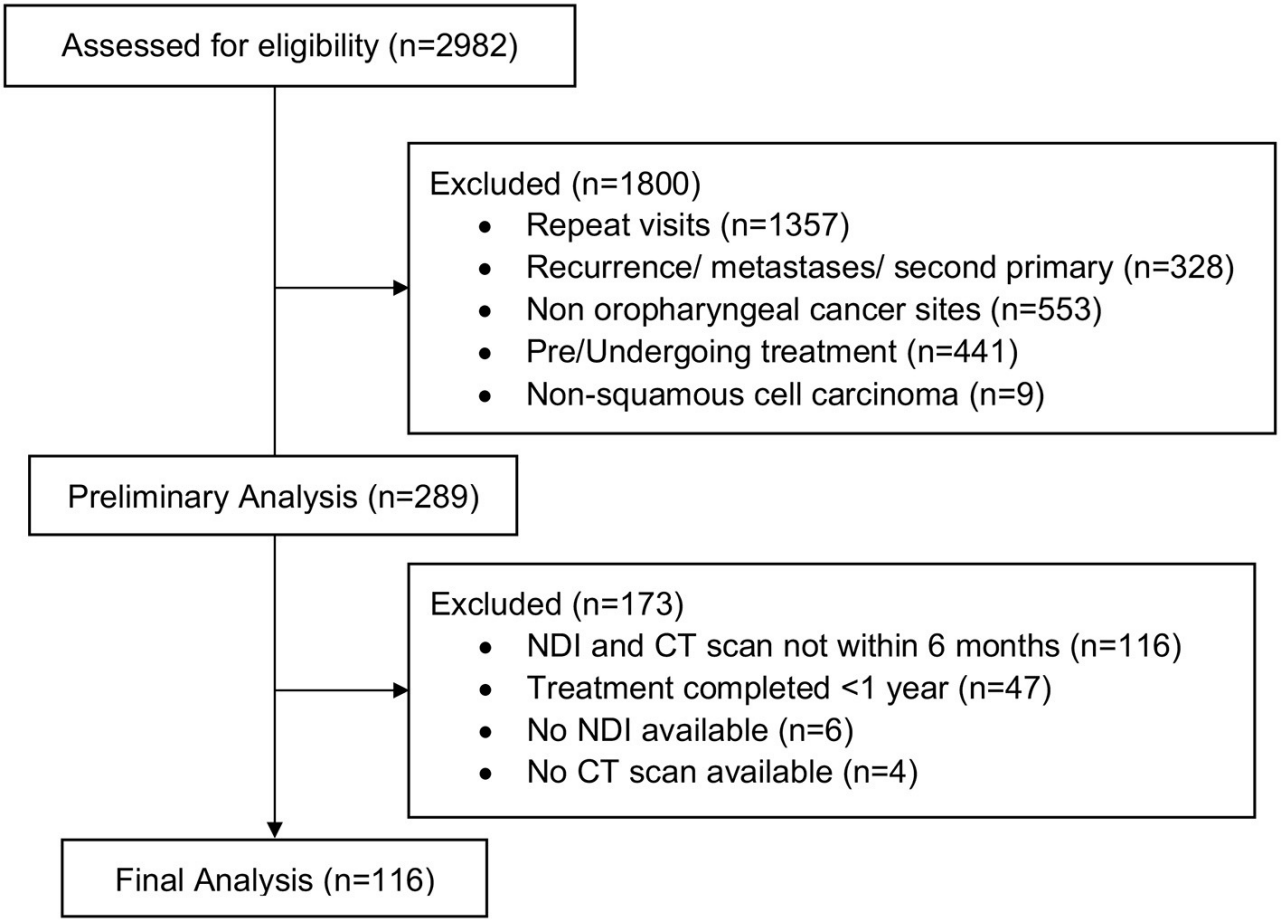
Consort diagram.

A total of 116, predominantly male (*n* = 95, 81.9%), survivors of oropharyngeal carcinomas (age 62.8 ± 8.2, 43.8–81.4) seen at the HNC survivorship clinic were included in this study as seen in the CONSORT diagram in Figure 2. Most survivors had advanced disease (stage III-Iva) (*n* = 71, 61.2%) with treatment modalities including surgery (*n* = 67, 57.7%), chemotherapy (*n* = 90, 77.6%), and radiation therapy (*n* = 112, 96.6%). Neck disability was categorized into three groups based on NDI scores: 1) no disability (*n* = 51, 44.0%), 2), mild (*n* = 46, 39.7%), and moderate disability (*n* = 19, 16.4%), and the time from treatment to clinic visit was an average of 3.1 ± 2.7 years (range 1.1–13.4). Demographics and clinical characteristics are summarized in Table 1.

**Table 1:** Demographic and clinical characteristics (*n* = 116).

**Variables**	***M* ±*SD***	***n* (%)**
Age, y	62.8 ± 8.2	
Male		95 (81.9)
Married		79 (68.1)
**Race**		
White		107 (92.2)
African American		8 (6.9)
**AJCC Stage**		
I–II		39 (33.6)
III–IV		71 (61.2)
**HPV Status**		
Positive		92 (79.3)
Negative		6 (5.2)
Unknown		18 (15.5)
**Neck Disability**		
None		51 (44.0)
Mild		46 (39.7)
Moderate or Higher		19 (16.4)
**Treatment Modality**		
Received Surgery		67 (57.8)
Received Chemotherapy		90 (77.6)
Received Radiation		112 (96.6)
Time since Treatment Completion, y	3.1 ± 2.7	

Univariable regression showed that there was a significant, inverse relationship between NDI scores and cervical disc degeneration score (*p* = 0.042) and age (*p* < 0.001). Univariable analyses are shown in Table 2. The multivariable regression model included age, time since treatment completion, whether the survivor had received surgical intervention, and CT findings of disc and facet degeneration. Multivariable analysis revealed a significant association between NDI score and age (−0.235, 95%CI [−0.395, −0.076], *p* = 0.004) and cervical disc degeneration (−1.461, 95%CI [−2.859, −0.064], *p* = 0.041) [*F*_(5)_ = 4.62, *p* < 0.001]. No significant association was shown between NDI and time since treatment completion (*p* = 0.483), history of surgical intervention (*p* = 0.525), or facet degeneration (*p* = 0.765). The explained variance of the NDI by these variables was 18.2%. The results of the multivariable linear regression are shown in Table 3.

**Table 2:** Univariable regression between neck disability scores and clinical and demographic characteristics (*n* = 116).

	**Neck Disability Scores**		***p*-value^a^**
	**M ±SD**	**Coefficient (95% CI)**	
**Age, y**		−0.30 (−0.444, −0.155)	0.000
**Time since Treatment Completion, y**		0.001 (−0.001, 0.001)	0.815
**Sex**			
Female	5.7 ± 5.9	(Base)	
Male	6.9 ± 7.1	1.24 (−2.06, 4.54)	0.459
**Marital Status**			
Unmarried	7.0 ± 7.6	(Base)	0.413
Married	6.1 ± 6.2	−0.60 (−0.85, 2.05)	
**Race**			
African American	9.8 ± 12.4	(Base)	0.189
White	6.4 ± 6.4	−3.34 (−1.66, 8.34)	
**HPV Status**			
Positive	6.6 ± 6.5	(Base)	
Negative	4.0 ± 4.4	−2.90 (−8.66, 2.85)	0.566
Unknown	5.7 ± 5.0	−1.19 (−6.54, 4.17)	
**AJCC Staging**			
I–II	6.9 ± 8.2	(Base)	0.861
III–IV	6.7 ± 6.4	−0.25 (−3.04, 2.55)	
**Treatment Modality**			
Surgery			
No	5.9 ± 6.3	(Base)	
Yes	7.3 ± 7.3	1.36 (−1.21, 3.92)	0.297
Chemotherapy			
No	5.7 ± 7.8	(Base)	
Yes	7.0 ± 6.6	1.42 (−4.89, 7.73)	0.653
Radiation Therapy			
No	13.0 ± 16.1	(Base)	
Yes	6.5 ± 6.4	−6.55 (−13.42, 0.33)	0.062
**CT Evaluation**
Disc (range 1–5)		−2.06 (−3.21, −0.91)	0.001
Facet (range 1–5)		−1.30 (−2.56, −0.045)	0.042

a *p-value according to Linear Regression Model and Likelihood Ratio Test; significance level at p < 0.05*.

**Table 3:** Results of the multiple linear regression analysis (*n* = 116) (*F*_5_ = 4.62, *p* < 0.001).

**Variables**	***Coefficient (95% CI)***	***p-value***
**Age, y**	−0.235 (−0.395, −0.076)	0.004
**Time since treatment completion, y**	0.000 (−0.001, 0.002)	0.483
**Surgical intervention**		
Yes	0.776 (−1.636, 3.188)	0.525
No	(Base)	
**CT Findings**		
Disc	−1.461 (−2.859, −0.064)	0.041
Facet	0.190 (−1.254, 1.633)	0.765

## Discussion

Treatments for HNC including surgical resection, reconstruction, and chemoradiation alone or in combination are intensive therapies often resulting in acute- and late-term functional and cosmetic deficits for the survivor population. Prior reports have shown neck disability affects up to three-quarters of post-treatment survivors, although identifying these individuals is not always straightforward. PROs have become increasingly important in survivorship initiatives, quickly identifying patients with higher degree and severity of disability so that early intervention may be provided to address symptoms. The neck disability index is one PRO which has been shown to correlate with decreased cervical range of motion and velocity, although the pathology of the functional deficit and reported disability are not fully elucidated. Our results show that aging and CT findings of cervical degeneration, osteoarthritis or disc disease, do not explain neck disability in the HNC survivor population, further supporting the idea that neck disability is secondary to treatment-related toxicity.

Our study found that advanced age at diagnosis and the presence of cervical degeneration are independent predictors of reduced neck disability. In non-cancer populations, neck disability is associated with advanced age, injury, and disc degeneration (29); however, our results suggest that the presence of cervical degeneration, either injury or age-related, is not sufficient to explain the disability reported by oropharyngeal survivors. If fact, only 18.2% of the variance in the NDI was explained by age, cervical degeneration, time since treatment, and the presence of surgical intervention. Examination into the effects of treatment on sarcopenia, fibrosis, and nerve damage may yield more information on the cause and pathophysiology of this disability. A study by Gane et al. showed similarly that advanced age was associated with reduced neck disability in a head and neck cancer population; however, their study specifically focused on those who had undergone surgery and neck dissections (8). We also found that neck disability was unrelated to surgical intervention but was more severe in the younger HNC population.

Cervical spine changes seen on CT were found to be associated with decreased neck disability. Peterson et al. found that the cervical spine degeneration was not associated with chronic neck pain or reported neck disability in those with and without trauma and similar findings have been demonstrated in the L-spine (30, 31). These findings may be due in part to those individuals with spinal degeneration adapting to limitations prior to treatment, thus reporting lower levels of disability. Additionally, those with cervical degeneration and with more advanced age may be less active than their younger counterparts, which would contribute to lower levels of perceived disability in those populations. While the cause of this relationship is speculative, our results show that age and age-related cervical arthropathy are inversely related to reported neck impairment in HNC survivors and the presence of neck pain or disability should not imply spinal degeneration in these patients when considering post-treatment physical therapy.

### Limitations

The survivors included in this study did not experience severe neck disability symptoms based on the NDI scoring, which limits the ability to interpret findings to those with mild to moderate disease. Additionally, the sample size was limited by availability of CT scans within 6 months of the NDI, limiting the ability to determine significance of the results or additional factors affecting neck disability. The majority of patients in our study received chemoradiation therapy, preventing analysis of the effects of non-surgical intervention or the effects of combinations of therapy on neck disability and CT findings.

## Conclusions

Neck disability is a commonly recognized symptom in HNC survivors although little research has been done to evaluate the relationship between neck disability and radiologic imaging in patients. Our study shows that neck disability is not attributable to cervical osteoarthritis or disc disease, suggesting that neck disability is a side-effect of treatment further studies may expand upon radiographic findings to include examination of soft-tissue structures. For those individuals with HNC, proactive therapy, such as physical or occupational even before treatment initiation may help prevent negative quality-of-life outcomes associated with treatment-related toxicity regardless of treatment modality. Future research may further the understanding of the biologic components and additional clinical findings of neck disability in the survivor population.

## Data Availability Statement

The original contributions presented in the study are included in the article/supplementary material, further inquiries can be directed to the corresponding author/s.

## Ethics Statement

The studies involving human participants were reviewed and approved by University of Pittsburgh's Human Research Protection Office. Written informed consent for participation was not required for this study in accordance with the national legislation and the institutional requirements.

## Author Contributions

AH: analysis of the results and writing of the manuscript. BB: radiologic review and image selection. JL: statistical analysis and writing of the manuscript. SP: contributed to manuscript preparation and results interpretation. JJ: supervised project. MN: conceived the original idea, supervised project, and performed primary data collection. All authors contributed to the article and approved the submitted version.

## Funding

Use of REDCAP in this study was supported by the Clinical and Translational Sciences Institute at the University of Pittsburgh Grant Number UL1-TR-001857.

## Conflict of Interest

The authors declare that the research was conducted in the absence of any commercial or financial relationships that could be construed as a potential conflict of interest.

## Publisher's Note

All claims expressed in this article are solely those of the authors and do not necessarily represent those of their affiliated organizations, or those of the publisher, the editors and the reviewers. Any product that may be evaluated in this article, or claim that may be made by its manufacturer, is not guaranteed or endorsed by the publisher.
